# Fistuloclysis Improves Liver Function and Nutritional Status in Patients with High-Output Upper Enteric Fistula

**DOI:** 10.1155/2014/941514

**Published:** 2014-02-26

**Authors:** Yin Wu, Jianan Ren, Gefei Wang, Bo Zhou, Chao Ding, Guosheng Gu, Jun Chen, Song Liu, Jieshou Li

**Affiliations:** Department of Surgery, Jinling Hospital, Medical School of Nanjing University, 305 East Zhongshan Road, Nanjing 210002, China

## Abstract

*Background.* We aimed to determine the efficacy of fistuloclysis in patients with high-output upper enteric fistula (EF). *Methods.* Patients were assigned into the fistuloclysis group (*n* = 35, receiving fistuloclysis plus total enteral nutrition (TEN)) and the control group (*n* = 60, receiving TEN). Laboratory variables were measured during the four-week treatment. *Results.* At baseline, variables were similar between the two groups. Delta value was defined as the changes from baseline to day 28. Compared with the control group, the fistuloclysis group showed greater improvements in liver function (Delta total bilirubin (TB): 20.3 ± 9.7 in the fistuloclysis group versus 15.6 ± 6.3 in the control group, *P* = 0.040; Delta direct bilirubin (DB): 12.5 ± 3.4 versus 10.0 ± 3.6, *P* = 0.011; Delta alkaline phosphatase (ALP): 98.4 ± 33.5 versus 57.6 ± 20.9, *P* < 0.001); nutritional status (Delta total protein: 21.8 ± 8.7 versus 10.7 ± 2.1, *P* < 0.001; Delta albumin: 11.3 ± 2.5 versus 4.2 ± 1.3, *P* < 0.001). In the fistuloclysis subgroups, biliary fistula patients had the maximum number of variables with the greatest improvements. *Conclusions.* Fistuloclysis improved hepatic and nutritional parameters in patients with high-output upper EF, particularly in biliary fistula patients.

## 1. Background

Enteric fistula (EF) is an abnormal communication between the enteric tract and surrounding tissues, involving leakage of bowel contents. Contamination of the abdominal cavity with enterogenic microorganisms and toxins stimulates production of inflammatory cytokines, leading to risk of multiple organ dysfunction syndrome and death [1]. EF with effluent more than 500 mL per day is considered as high-output EF, which commonly occurs in patients with upper EF [2]. Patients with high-output upper EF are at high risk of malnutrition, wound infection, and coexisting sepsis. Despite the advances in the supportive care, the mortality rates still remain at 5% to 20% [3]. In addition, EF leads to patients' anxiety, loss of self-esteem, and financial hardship [4]. Therefore, high-output upper EF represents a considerable challenge to physicians, patients, and families.

Conventional treatments include source control, sepsis treatment, abdominal lavage and drainage, antibiotics, nutritional therapy, and definitive operations [5]. In patients with high-output upper EF, persistent enteric fluid loss and catabolism status result in malnutrition, electrolyte imbalance, and organ dysfunctions. Nutritional management remains a key mainstay [6]. It was the introduction of nutritional therapy that markedly reduced the mortality of EF from 100% to 11% [5]. Nevertheless, the nutritional management of patients with high-output upper EF is still challenging. Parenteral nutrition (PN) is the first choice for this population [7]. Unfortunately, the complications of PN (such as liver dysfunction, catheter-related infections, and hyperglycemia) are common and severe [8]. It is advisable that enteral nutrition (EN) should be applied as soon as possible, but EN has the disadvantage of increasing the volume of fistula output further [9]. The technique called “fistuloclysis combined with TEN” is defined as the output collected from the proximal fistula is reinfused into the distal fistula through a feeding tube, while TEN is infused simultaneously through this tube or another nasojejunal tube. This technique could provide nutrition without PN. Although the technique is only feasible for a selected population, it provides an alternative choice [10].

Current researches regarding fistuloclysis are primarily in the form of case reports or case series with a small cohort [11]. The precise mechanisms of fistuloclysis remain to be elucidated. We evaluated the efficacy of fistuloclysis by comparing two cohorts with and without fistuloclysis, hoping to optimize the nutritional management. To the best of our knowledge, it is the first study to determine the efficacy of fistuloclysis in terms of laboratory and clinical variables in patients with high-output upper EF.

## 2. Methods

### 2.1. Study Design

This was a retrospective study of patients admitted to the Department of Surgery, Jinling Hospital in China. This facility included 37 beds with about 1500 admissions each year. As a national center for gastrointestinal disorders, we annually serve approximately 400 patients with various fistulas. The treatment protocol was approved by the Jinling Hospital Ethical Subcommittee, with written informed consent obtained from patients or their relatives. All research works with humans were in compliance with the Helsinki Declaration.

### 2.2. Patient Population

Between January 1, 2001, and October 6, 2013, a total of 528 patients had high-output upper EF. The enrollment criteria included the following: (1) ≥18 years old and ≤65 years old; (2) gastrointestinal fistula confirmed by CT scan or gastroenterography; (3) daily fistula output in excess of 500 mL; (4) intact small intestine longer than 100 cm and recovered function (indicated by reduced nasogastric aspirate and active stoma output); (5) exclusion of obstructions in the distal bowel.

Exclusion criteria included the following: (1) previous renal diseases (serum creatinine ≥ 0.5 *μ*mol/L) or liver diseases (serum total bilirubin ≥ 20.5 *μ*mol/L); (2) lactational or gestational period; (3) leucopenia; (4) confirmed immunodeficiency or coagulation disorders; (5) missing laboratory values; (6) TEN or TEN along with fistuloclysis treatments for less than 28 days; (7) loss of followup within one year after discharge.

According to the exclusion criteria, 95 patients were eligible and finally enrolled. For all patients, TEN was applied once the intra-abdominal sepsis had been controlled [12]. Patients were assigned into the fistuloclysis group (*n* = 35, receiving fistuloclysis plus TEN) and the control group (*n* = 60, receiving TEN alone).

In the fistuloclysis group, patients were further divided into three subgroups according to the EF locations: Group 1, patients with jejunal-ileal fistula (*n* = 11); Group 2, patients with biliary fistula (*n* = 16); Group 3, patients with duodenal fistula (*n* = 8).

In the control group, only TEN was utilized due to following reasons: (1) in certain cases,where there were deep EFs, continuous irrigation-suction was applied through double-lumen tubes. The output from the proximal fistula was mixed with irrigation fluid, making it unavailable for reinfusion [13]; (2) they refused the technique due to personal reasons (3); the technique was not widely applied in our hospital before March 1, 2005.

### 2.3. Management of EF

For EF management, the four pivotal principles consisted of correction of intravascular volume deficit, drainage of abscess, control of fistula output, and protection of the skin. The EF-associated abdominal sepsis was treated according to standards: (1) adjustment for fluid and electrolyte imbalance; (2) norepinephrine (intravenously, 10 *μ*g/min, 3 h) as primary vasopressor; (3) early goal-directed therapy (MAP > 65 mmHg, and SvO_2_ > 65%); (4) source control by surgical or percutaneous drainage; (5) antibiotics therapy. Mechanical ventilation (adaptive support, controlled mechanical, intermittent mandatory, etc.) and other assistant treatments were applied as needed. Hyperglycemia was controlled with short-acting insulin analogs [14].

### 2.4. Techniques of Fistuloclysis

The fistula was intubated with a MIC-G balloon retention gastrostomy tube (Vygon, Cirencester, UK) (for patients with biliary fistula, a T-tube was applied). The output from the proximal stoma was collected with a triple catheterization cannula which was connected to aspiration pumps at a negative pressure of 150 to 200 millibars. The freshly collected succus entericus or bile was drained into a sterile bag and reinfused back to the distal limb of the fistula through a Foley catheter at specific rate in accordance with the stoma output.

### 2.5. Nutritional Therapy

The feeding goal was at least 30 kcal/kg^−1^/d^−1^ and 1.5 to 2 g of protein/kg^−1^/d^−1^, while 2 g of nitrogen for per liter of fistula output was administered. High-output EF required 1.5–2 times the usual calories because of the ongoing loss [15]. All patients received total parenteral nutrition (TPN) (all in one fluid, Jinling Hospital, Nanjing, China) for varying intervals before fistuloclysis/TEN to induce stability. Subsequently, in the fistuloclysis group, TEN and the collected succus entericus were infused into the distal fistula through the Foley catheter or through the nasojejunal tube (for patients with biliary fistula) for at least 28 days. If patients did not tolerate nonelemental feed (Ensure powder (Abbot, Shanghai, China), Nutrison (Nutricia, Shanghai, China)), then they changed to receive semielemental feed (Perative, Abbot, Shanghai, China) or elemental feed (Peptisorb, Abbot, Shanghai, China). In the control group, only TEN was applied for at least 28 days through similar ways as the fistuloclysis group. The fistula output was drained out through double-lumen tubes [13] and discarded, while the volume deficit was corrected by fluid replacement.

### 2.6. Laboratory Parameters Assessments

The variables were retrospectively collected from the patient's electronic health record. For each enrolled patient, laboratory variables were collected before fistuloclysis/TEN (baseline) and on days 14 and 28 after fistuloclysis/TEN. Venous blood in all laboratory tests was drawn between 5 a.m. and 6 a.m. in the morning, and laboratory values were calculated within 2 hours after blood collection.

Laboratory tests recorded for study purpose included the following: hepatic function indexes (serum total bilirubin (TB), direct bilirubin (DB), indirect bilirubin (IB), *γ*-glutamyltransferase (*γ*-GGT), alkaline phosphatase (ALP), alanine aminotransferase (ALT), total protein (TP), and albumin (Alb)) and other routine laboratory parameters (white blood cells (WBC), C-reactive protein (CRP), prothrombin time (PT), activated partial thromboplastin time (APTT), red blood cells (RBC), and hemoglobin (Hb)). Missing laboratory values were imputed with the closest value of the previous or following day when available, within a 48-hour window.

The standard values of laboratory tests in our hospital were as follows: TB, 0–19 *μ*mol/L; DB, 0–6.8 *μ*mol/L; IB, 0–12.2 *μ*mol/L; *γ*-GGT, <50 U/L; ALP, 30–120 U/L; ALT, 2–50 U/L; TP, 64–83 g/L; Alb, 35–55 g/L; WBC, 3.9–10 × 10^9^/L; CRP, <8 mg/L; PT, 11–13.7 s; APTT, 20–40 s; RBC, 4–5.5 × 10^12^/L; Hb, 120–160 g/L.

### 2.7. Observing Severity Scores

Routine examinations, blood gas analysis, drug administrations (types of vasopressor, dosage, etc.), and culture results (blood, stool, and urine) were monitored after admission. The acute physiology and chronic health evaluation II (APACHE II) score and sequential organ failure assessment (SOFA) score were calculated for all patients [12, 13]. The fistula output was measured daily. Patients' demographics, diagnosis, and adverse events related to fistuloclysis were also recorded. Patients were followed up through visits or telephone calls for one year after discharge.

### 2.8. Statistical Analysis

The Kolmogorov-Smirnov test was applied to check for normal distribution. Normal distributed variables were compared using variance or *t*-test. Wilcoxon rank-sum test or Mann-Whitney *U* test was applied for nonnormally distributed variables. Continuous variables were presented as means ± standard deviation (SD).

Delta value was defined as the changes from baseline to day 28 for each variable. The significance of differences in variables (the baseline value and the delta value, resp.) between the fistuloclysis group and the control group was compared using the independent-samples *t*-test. To estimate the time-dependent changes of variables within groups, the one-way ANOVA was employed. One-year survival curves were compared using the Kaplan-Meier method and Log-rank test. All tests were two-sided, and a *P* value < 0.05 was deemed significant. Statistical analyses and plots were performed using SPSS Software (version 18.0; SPSS Inc., Chicago, IL).

## 3. Results

### 3.1. General Characteristics of Patients

**Figure 1:**
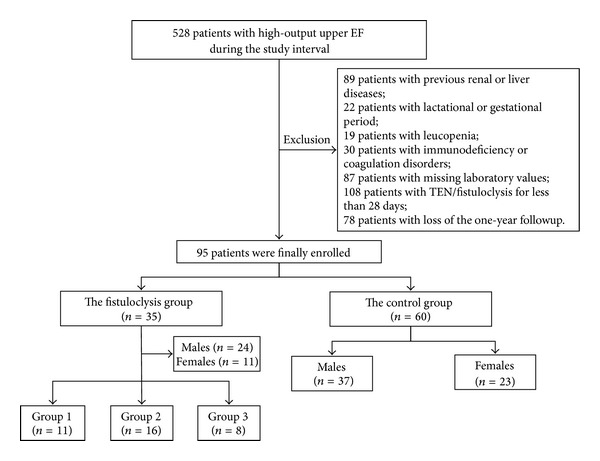
Flow chart of the study design. A total of 528 patients were enrolled during the interval of this study. According to the exclusion criteria, 95 patients were eligible and finally enrolled. Patients were assigned into the fistuloclysis group (*n* = 35, receiving fistuloclysis plus total enteral nutrition (TEN)) and the control group (*n* = 60, receiving TEN only). The fistuloclysis group was divided into subgroups according to EF locations: Group 1: patients with small intestinal fistula (*n* = 11); Group 2: patients with biliary fistula (*n* = 16); Group 3: patients with duodenum fistula (*n* = 8).

During the interval of this study, a total of 528 patients had high-output upper EF. According to the exclusion criteria, 95 patients were eligible and were assigned into the fistuloclysis group (*n* = 35, 24 males and 11 females) and the control group (*n* = 60, 37 males and 23 females) (Figure 1). Patients' demographics are summarized in Table 1. There were no significant differences with respect to age, gender, BMI, and severity scores between the two groups. Additionally, no significant differences in terms of etiology and underlying disease could be detected. Notably, trauma was listed as the top etiology for both groups (40.0% in the fistuloclysis group; 36.7% in the control group, resp.). Significant differences could be detected in fistula locations (biliary fistula, *P* = 0.042; duodenal fistula, *P* = 0.033). Moreover, hospital charges ($44,261.3 ± $5,631.2 versus $30,215.1  ±  6,518.9, *P* < 0.001), hospital stay (74.6  ±  19.8 versus 56.2 ± 24.3, *P* < 0.001), and hospital mortality (8.3% versus 0.0%, *P* = 0.025) were significantly higher in the control group compared with the fistuloclysis group.

In the fistuloclysis group, patients were further divided into Group 1 (*n* = 11, 7 males and 4 females), Group 2 (*n* = 16, 11 males and 5 females), and Group 3 (*n* = 8, 5 males and 3 females) according to their fistula locations. Patients' demographics are summarized in Table 2. There were no significant differences in terms of age, gender, BMI, severity scores, etiology, and underlying disease between the three subgroups.

### 3.2. Comparisons of Variables in the Fistuloclysis Group and the Control Group

Using the independent-samples *t*-test, we compared the laboratory variables in the fistuloclysis group and the control group, as summarized in Table 3. At baseline, all variables were statistically identical between the two groups except for CRP (*P* = 0.004). Patients in both groups had a decline tendency in hepatic indexes over time, from baseline to day 28, in order of the fistuloclysis group and the control group: TB (from 38.2 ± 9.8 to 18.1 ± 6.2, *P* < 0.001 versus from 40.7 ± 11.2 to 24.8 ± 7.1, *P* = 0.013); DB (from 19.6 ± 8.5 to 7.2 ± 2.3, *P* < 0.001 versus from 21.4 ± 10.0 to 11.3 ± 3.5, *P* = 0.012); IB (from 18.3 ± 6.6 to 12.6 ± 3.6, *P* = 0.026 versus from 19.1 ± 7.1 to 13.2 ± 4.4, *P* = 0.043); ALP (from 212.2 ± 85.1 to 113.7 ± 41.3, *P* < 0.001 versus from 219.7 ± 89.9 to 161.2 ± 34.7, *P* = 0.005); GGT (from 210.9 ± 90.1 to 53.4 ± 23.9, *P* < 0.001 versus from 223.4 ± 62.4 to 103.7 ± 37.5, *P* < 0.001). In the fistuloclysis group, a persistent decline trend was also observed in the volume of fistula output (from 1,306.2 ± 281.1 to 519.6 ± 173.2, *P* < 0.001).

Compared with baseline, patients in both groups exhibited a significantly time-dependent increasing trend in nutritional parameters, from baseline to day 28, in order of the fistuloclysis group and the control group: TP (from 56.7 ± 16.6 to 78.5 ± 25.1, *P* < 0.001 versus from 58.9 ± 11.8 to 69.7 ± 28.4,  *P* = 0.032); Alb (from 31.2 ± 3.4 to 42.5 ± 12.7, *P* < 0.001 versus from 32.5 ± 12.8 to 36.8 ± 7.6, *P* = 0.078). For all of the patients, there were no significant alterations with respect to WBC, CRP, PT, APTT, RBC, and Hb during the observation period (data not shown).

Delta value was defined as the changes from baseline to day 28 for each variable. Compared with the control group, the fistuloclysis group showed greater improvements in liver function (Delta TB: 20.3 ± 9.7 in the fistuloclysis group versus 15.6 ± 6.3 in the control group (the same sequence thereafter), *P* = 0.040; Delta DB: 12.5 ± 3.4 versus 10.0 ± 3.6, *P* = 0.011; Delta ALP: 98.4 ± 33.5 versus 57.6 ± 20.9, *P* < 0.001; Delta GGT: 157.5 ± 52.6 versus 119.8 ± 38.5, *P* = 0.019), nutritional status (Delta TP: 21.8 ± 8.7 versus 10.7 ± 2.1, *P* < 0.001; Delta Alb: 11.3 ± 2.5 versus 4.2 ± 1.3, *P* < 0.001), and the volume of fistula output (Delta value: 786.6 ± 285.8 versus 391.5 ± 125.3, *P* < 0.001).

### 3.3. Comparisons of Fistuloclysis Efficacy in Patients with Different EF Locations

Another main objective of the study was to evaluate the efficacy of fistuloclysis in regard to EF locations. We calculated changes from baseline to day 28 for each variable in the fistuloclysis subgroups. The significance of difference was compared by means of one-way ANOVA. The results are shown in Tables 4 and 5. For all of the fistuloclysis subgroups, the greatest attenuations in TB (*P* = 0.011), DB (*P* = 0.040), IB (*P* < 0.001), and ALP (*P* = 0.022) were observed in Group 2 patients. Additionally, the greatest elevations in TP (*P* = 0.005) and Alb (*P* = 0.020) were observed in Group 2 patients. By contrast, Group 1 patients produced the least changes in terms of TB (*P* = 0.011), DB (*P* = 0.040), IB (*P* < 0.001), ALP (*P* = 0.022), TP (*P* = 0.005), and Alb (*P* = 0.020). No significant differences were detected with respect to GGT, WBC, CRP, fistula output, PT, APTT, RBC, and Hb between the three subgroups (data not shown). Cumulatively, Group 2 patients had the maximum number of variables with the greatest improvements.

### 3.4. One-Year Survival Rate and Survival Time

**Figure 2:**
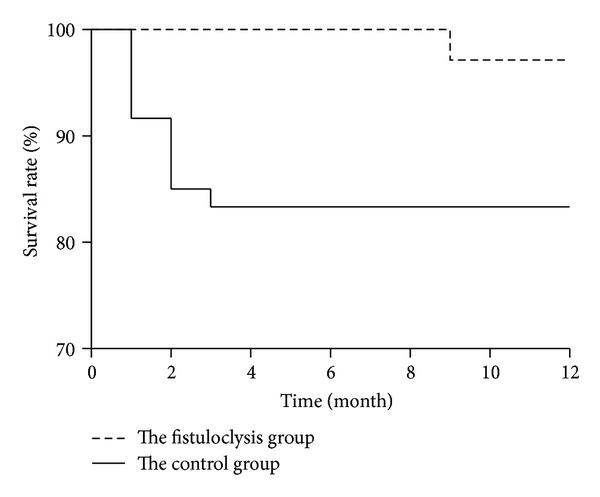
Kaplan-Meier analysis of one-year survival rate and survival time. During the one-year followup, one patient (2.9%) in the fistuloclysis group died, whereas ten patients (16.7%) in the control group died. The numbers of patients remaining in each group are shown in parentheses. The Log-rank test demonstrated the superiority of fistuloclysis compared with the control group (*P* = 0.045).

Figure 2 illustrates the results of Kaplan-Meier analysis for the fistuloclysis group and the control group. One patient (2.9%) in the fistuloclysis group died, whereas ten patients (16.7%) in the control group died within the one-year followup. With respect to survival time, the Log-rank test demonstrated the superiority of fistuloclysis plus TEN as compared with TEN alone (*P* = 0.045).

### 3.5. Adverse Events

Safety and tolerability assessment of fistuloclysis included diarrhea, vomiting, nausea, abdominal pain, and abdominal distension. A total of seventy-two patients with high-output upper EF received fistuloclysis, whereas ten cases withdrew due to intolerance. They had fistuloclysis for not more than ten days and gave up this technique due to complaints of diarrhea, nausea, vomiting, and abdominal dull pain. The adverse events resolved soon after discontinuation of fistuloclysis. Twenty-seven patients were excluded according to the exclusion criteria, leaving thirty-five patients in the fistuloclysis group. In Group 1, one patient (9.1%) developed mild diarrhea. In Group 2, two patients (12.5%) developed mild nausea and vomiting. In Group 3, one patient (12.5%) developed abdominal dull pain. The adverse events all relieved later during the period of fistuloclysis.

## 4. Discussion

In this study, we demonstrated fistuloclysis as an effective and safe technique in patients with high-output upper EF, especially in patients with biliary fistula. These benefits may be due to improved liver function and nutritional status, along with inhibition of fistula output. To the best of our knowledge, this is the largest comparison trial of fistuloclysis in terms of laboratory and clinical variables.

In patients with high-output upper EF, malnutrition is the leading cause of mortality. Inadequate nutrition intake due to restricted intake, increased nutrition requirements related to sepsis and catabolism, and nutrient losses through the fistula output all contribute to malnutrition [16]. It is recommended that definitive surgery be delayed for at least three months from the last surgery. Therefore, long-term and effective nutrition support plays a pivotal role in preparing patients qualified for surgery [17].

PN is regarded as the standard care for patients with high-output EF. PN combined with somatostatin could decrease gastrointestinal secretions by 30–50%, which makes this regimen popular among this population [17]. However, PN has many potential complications, including acute liver dysfunction, atrophy of intestinal villus, bacteria translocation, catheter-related infections, and hyperglycemia. Specifically, liver dysfunction is fairly common, occurring in 15%–40% of patients [18]. Moreover, PN is expensive and highly demanding of professional nursing care. The somatostatin was also reported to be detrimental by reducing splanchnic blood flow and nutrient absorption [19]. EN, as opposed to PN, has been shown to improve intestinal barrier function, reduce the risk of bacterial translocation, and attenuate liver dysfunction [20]. EN is effective in downregulating inflammation response, reducing oxidative stress, and improving outcomes [21]. In our center, patients began to receive EN as soon as they could tolerate it. However, EN has the disadvantage of increasing the volume of fistula output [9]. Therefore, the conventional routes of nutrient delivery are hard to establish in patients with high-output upper EF.

Fistuloclysis has been developed for decades. Teubner et al. found that fistuloclysis combined with TEN could replace PN by increasing body weight and serum albumin levels as well as decreasing the length of hospital stay [12]. Several case series reported successful applications of this technique [22]. The utilization of fistuloclysis has several advantages.

First, fistuloclysis improves liver function better when compared with TEN alone. All patients received TPN before fistuloclysis/TEN for adaptation. TPN is associated with impairment of biliary secretion and cholestasis [23], which were evidenced by dramatically increased serum TB, DB, IB, ALP, and GGT levels in patients at baseline. In addition, dehydration and malnutrition also lead to cholestasis [24, 25]. Cumulatively, patients with high-output upper EF are at high risk of cholestasis. Elevated serum bilirubin impairs the bactericidal activity of neutrophils, leukocyte migration, and complement-dependent reactions, leading to diminished immune function and poor prognosis [26]. In the current study, we observed attenuated cholestasis in both groups after four-week fistuloclysis/TEN, which matches previous findings showing that reversal of cholestasis could only occur after discontinuation of PN and establishment of EN [27]. It is noteworthy that cholestasis relieved more dramatically and rapidly in the fistuloclysis group compared with the control group. As a predominant gastrointestinal hormone, cholecystokinin (CCK) might partly explain this phenomenon. CCK is produced by endocrine cells in the duodenum and upper jejunum, stimulating gallbladder contraction and bile secretion. Daily infusions of CCK attenuated the PN-associated cholestasis [28]. The stimuli for secretion of CCK are the presence of fats, proteins, and amino acids in the duodenum [29]. Another CCK-releasing factor is a trypsin-sensitive monitor peptide which resides in the upper intestinal lumen. But it might be lost through the fistula output. Previous researches demonstrated that rapid perfusion of the upper intestine with saline would wash out this peptide and inhibit CCK secretion [30]. Therefore, the intact digestive process reconstructed by fistuloclysis could stimulate CCK secretion and further alleviate cholestasis.

Second, fistuloclysis provides essential enzymes and bile acids for optimal utilization of EN [31]. EN has numerous benefits such as anti-inflammation, decreasing infection risk, reducing oxidative stress, and preventing liver dysfunction [20]. The fistula fluid contains an array of enzymes, including salivary amylase, gastric pepsin, and pancreatic enzymes. It is superior to artificial products, since it has optimal pH value for activating proenzymes and appropriate enzyme components for efficient utilization of EN [32]. Bile acids are synthesized in the liver and secreted into the duodenum and intestine. At the terminal ileum, about ninety-five percent of bile acids are recycled to the liver through the enterohepatic circulation [33]. However, the recycling process was disturbed in patients with upper EF. A lack of bile acids inhibits the complete absorption of fatty acids, phospholipids, and fat-soluble vitamins, leading to complications of cholestasis and diarrhea. The enterohepatic circulation could be reconstructed by fistuloclysis, thus promoting nutritional absorption. We observed that serum TP and albumin levels increased to a greater extent in the fistuloclysis group than in the control group. The improved nutritional status might contribute to the higher survival rate of the fistuloclysis group. This finding was consistent with a previous study showing 42% mortality rate in patients with serum albumin of <2.5 g/dL compared to zero mortality in patients with albumin > 3.5 g/dL [34]. We also indicated that fistuloclysis inhibited CRP levels, although not to a significant extent. As an acute-phase protein, serum CRP levels reflect the inflammation response and stress status [35]. The decline tendency of CRP levels may be partly due to the anti-inflammation and anti-infection effects of EN.

Third, fistuloclysis has the capacity to decrease fistula output when compared with TEN alone. It is estimated that eight to ten liters of fluid is released by upper gastrointestinal glands each day, while ninety-eight percent of the fluid is reabsorbed near the ileocecal valve. High-output upper EF is associated with excessive loss of fluid and electrolytes, cumulating in hyponatremia, hypochloremia, hypokalemia, metabolic acidosis/alkalosis, and renal dysfunction [17]. It is difficult to maintain fluid and electrolyte balance in this population, since fluid replacement with large volume would aggravate tissue hypoperfusion, heart burdens, and cerebral edema [36]. We observed that the fistula output decreased significantly in the fistuloclysis group, which is in line with a previous study showing an apparent inhibition of upper gastrointestinal digestive secretions by fistuloclysis [10]. The mechanisms still remain obscure. We speculated that this phenomenon might be attributed to restoration of bowel continuity and physiological digestive process.

Fourth, fistuloclysis is associated with cost savings. We proved that, compared with the control group, the fistuloclysis group had lower hospital charges and shorter hospital stay, which might be due to fewer complications and better prognosis of this group. On the other hand, EN combined with fistuloclysis relieves financial burdens compared with PN (EN is 4 to 12.5 times cheaper than PN). The training period for fistuloclysis (less than 2 weeks) is shorter than that for home PN (usually 4–6 weeks) [37], which potentializes the possibility of home care and reduces length of hospital stay [12]. The cost savings would be greater if the labor intensity and nursing time are taken into account. The economic benefit of fistuloclysis makes it particularly suitable in facilities with limited medical resources.

It remains unclear why patients with biliary fistula exhibited the best therapeutic efficacy of fistuloclysis. Anatomic locations affect not only the effluent volume, but also the components of fistula output. Compared with other fistula patients, biliary fistula patients lost less succus components and had longer intact gastrointestinal tract. Notably, the reinfused fluid of biliary fistula patients had the highest concentrations of bile salts, which were presumed to play a predominant role in the fistuloclysis. As a retrospective study, we did not run tests on the succus. Future studies are needed to analyze the fistula output to determine the efficacious components.

Fistuloclysis utilization may produce several problems, including tube dislodgement-associated underfeeding and effluent leakage-related skin corrosion. A rare complication is a “swallowed” feeding tube, which is internalized by peristaltic activity of the distal intestine [12]. It is not known whether the feeding tube delays the spontaneous healing of fistula. Moreover, fistuloclysis has not been widely applied in America, partly due to aesthetic concerns [37].

Our study has several limitations, which must be acknowledged. First, the volume of reinfused output varied among patients, making this study a qualitative but not a quantitative experiment. Second, given the retrospective nature, there are possibilities of bias and inaccurate data collections. After excluding a large amount of patients strictly according to the exclusion criteria, accurate data collections were confirmed. Ideally, a prospective randomized controlled trial should be conducted but with the difficulty of patients' agreements. Notably, our center is applying for a prospective multicentral clinical trial in terms of fistuloclysis. This current research aimed to introduce this technique to a broader audience, hoping to optimize the nutritional management as soon as possible. Third, some pivotal nutritional parameters, such as fibronectin, transferrin, and prealbumin, were not measured. A possible influence of fistuloclysis on these unmeasured variables cannot be excluded. Finally, the relatively small sample size and higher rates of males would depress the statistical power.

## 5. Conclusion

By focusing on a targeted population, we confirmed a pivotal role of fistuloclysis. Fistuloclysis appeared to be an effective and safe technique for patients with high-output upper EF, particularly in biliary fistula patients. The efficacy might be attributed to improvements of liver function, optimization of EN absorption, and inhibition of fistula output. We recommended that patients with high-output upper EF be considered for fistuloclysis as soon as possible.

## Figures and Tables

**Table 1:** Demographics and clinical characteristics of the two groups.

Characteristics	Fistuloclysis (*n* = 35)	Control (*n* = 60)	*P* value
Age (years)	50.2 ± 14.1	55.8 ± 11.2	0.687
Male, *n* (%)	24 (68.6)	37 (61.7)	0.650
BMI (kg/m^2^)	19.1 ± 3.5	19.7 ± 2.3	0.627
Scores on admission			
APACHE II (24 h)	11.5 ± 3.0	12.2 ± 2.6	0.208
SOFA (24 h)	4.5 ± 1.2	4.8 ± 1.1	0.071
Etiology, *n* (%)			
Trauma	14 (40.0)	22 (36.7)	0.747
Tumor	3 (8.6)	7 (11.7)	0.637
Ischemic enteropathy	1 (2.9)	1 (1.7)	0.698
Operations	10 (28.6)	13 (21.7)	0.449
Pancreatitis	5 (14.3)	8 (13.3)	0.897
IBD	2 (5.7)	9 (15.0)	0.175
Fistula locations, *n* (%)			
Jejunal-ileal	11 (31.4)	20 (33.3)	0.849
Biliary	16 (45.7)	3 (5.0)	0.042*
Duodenal	8 (22.9)	37 (61.7)	0.033*
Underlying disease, *n* (%)			
Cancer	6 (17.1)	7 (11.7)	0.456
Cardiovascular disease	2 (5.7)	2 (3.3)	0.579
Diabetes	4 (11.4)	2 (3.3)	0.120
COPD	6 (17.1)	12 (20.0)	0.732
None	18 (51.4)	37 (61.7)	0.330
Charges ($), mean ± SD	30215.1 ± 6518.9	44261.3 ± 5631.2	<0.001*
Hospital stay (days), mean ± SD	56.2 ± 24.3	74.6 ± 19.8	<0.001*
Hospital mortality, *n* (%)	0 (0)	5 (8.3)	0.025*

BMI: body mass index; APACHE II: acute physiology score and chronic health evaluation II; SOFA: sequential organ failure assessment score; IBD: inflammatory bowel disease; COPD: chronic obstructive pulmonary disease. Data were presented as mean ± SD. *P* value < 0.05 was deemed significant.

**Table 2:** Demographics and clinical characteristics of the fistuloclysis subgroups.

Characteristics	Group 1 (*n* = 11)	Group 2 (*n* = 16)	Group 3 (*n* = 8)	*P* value
Age (years)	53.2 ± 14.1	48.9 ± 11.2	47.9 ± 13.4	0.346
Male, *n* (%)	7 (63.6)	11 (68.8)	5 (62.5)	0.680
BMI (kg/m^2^)	20.1 ± 3.5	19.5 ± 2.6	18.8 ± 4.2	0.362
Scores on admission				
APACHE II (24 h)	11.0 ± 3.1	12.2 ± 2.7	11.1 ± 3.5	0.072
SOFA (24 h)	4.4 ± 1.2	4.9 ± 1.1	4.4 ± 1.3	0.060
Etiology, *n* (%)				
Trauma	4 (36.4)	7 (43.8)	3 (37.5)	0.916
Tumor	1 (9.1)	1 (6.3)	1 (12.5)	0.876
Ischemic enteropathy	1 (9.1)	0 (0)	0 (0)	0.304
Operations	2 (18.2)	6 (37.5)	2 (25.0)	0.527
Pancreatitis	2 (18.2)	2 (12.5)	1 (12.5)	0.904
IBD	1 (9.1)	0 (0)	1 (12.5)	0.272
Underlying disease, *n* (%)				
Cancer	1 (9.1)	3 (18.8)	2 (25.0)	0.629
Cardiovascular disease	1 (9.1)	1 (6.3)	0 (0)	0.563
Diabetes	1 (9.1)	2 (12.5)	1 (12.5)	0.956
COPD	2 (18.2)	2 (12.5)	1 (12.5)	0.908
None	7 (63.6)	8 (50.0)	4 (50.0)	0.752

Group 1: patients with jejunal-ileal fistula; Group 2: patients with biliary fistula; Group 3: patients with duodenal fistulas. BMI: body mass index; APACHE II: acute physiology score and chronic health evaluation II; SOFA: sequential organ failure assessment score; IBD: inflammatory bowel disease; COPD: chronic obstructive pulmonary disease. Data were presented as mean ± SD. *P* value < 0.05 was deemed significant.

**Table 3:** Laboratory and clinical variables before and after 28-day treatments in the two groups.

Variables	Groups	Baseline	Day 14	Day 28	Delta	*P* ^a^ Value
TB (*μ*mol/L)	Fistuloclysis	38.2 ± 9.8	26.2 ± 8.5	18.1 ± 6.2	20.3 ± 9.7	<0.001*
	Control	40.7 ± 11.2	35.8 ± 10.6	24.8 ± 7.1	15.6 ± 6.3	0.013*
	*P* ^b^	0.243			0.040*	
DB (*μ*mol/L)	Fistuloclysis	19.6 ± 8.5	12.6 ± 5.8	7.2 ± 2.3	12.5 ± 3.4	<0.001*
	Control	21.4 ± 10.0	18.2 ± 7.5	11.3 ± 3.5	10.0 ± 3.6	0.012*
	*P* ^b^	0.139			0.011*	
IB (*μ*mol/L)	Fistuloclysis	18.3 ± 6.6	13.2 ± 4.3	12.6 ± 3.6	5.7 ± 2.0	0.026*
	Control	19.1 ± 7.1	17.7 ± 5.6	13.2 ± 4.4	5.9 ± 1.5	0.043*
	*P* ^b^	0.183			0.285	
ALT (U/L)	Fistuloclysis	55.6 ± 20.7	56.4 ± 22.3	48.1 ± 16.5	7.3 ± 3.1	0.025*
	Control	50.5 ± 17.6	62.5 ± 25.1	53.6 ± 15.7	3.2 ± 1.2	0.181
	*P* ^b^	0.058			0.033*	
ALP (U/L)	Fistuloclysis	212.2 ± 85.1	165.1 ± 65.2	113.7 ± 41.3	98.4 ± 33.5	<0.001*
	Control	219.7 ± 89.9	188.3 ± 69.5	161.2 ± 34.7	57.6 ± 20.9	0.005*
	*P* ^b^	0.182			<0.001*	
GGT (U/L)	Fistuloclysis	210.9 ± 90.1	106.2 ± 44.7	53.4 ± 23.9	157.5 ± 52.6	<0.001*
	Control	223.4 ± 62.4	177.2 ± 70.3	103.7 ± 37.5	119.8 ± 38.5	<0.001*
	*P* ^b^	0.422			0.019*	
TP (g/L)	Fistuloclysis	56.7 ± 16.6	65.2 ± 17.3	78.5 ± 25.1	21.8 ± 8.7	<0.001*
	Control	58.9 ± 11.8	62.1 ± 27.3	69.7 ± 28.4	10.7 ± 2.1	0.032*
	*P* ^b^	0.763			<0.001*	
Alb (g/L)	Fistuloclysis	31.2 ± 3.4	37.1 ± 9.5	42.5 ± 12.7	11.3 ± 2.5	<0.001*
	Control	32.5 ± 12.8	33.4 ± 10.5	36.8 ± 7.6	4.2 ± 1.3	0.078
	*P* ^b^	0.472			<0.001*	
WBC (10^9^/L)	Fistuloclysis	11.1 ± 4.0	10.3 ± 3.7	8.4 ± 2.2	2.6 ± 0.9	0.418
	Control	10.9 ± 3.9	10.1 ± 3.2	9.3 ± 2.4	1.8 ± 0.4	0.068
	*P* ^b^	0.304			0.061	
CRP (mg/L)	Fistuloclysis	12.6 ± 3.5	14.4 ± 6.1	10.1 ± 3.7	2.3 ± 1.1	0.073
	Control	15.2 ± 3.8	12.4 ± 4.9	13.8 ± 4.2	1.5 ± 0.5	0.081
	*P* ^b^	0.004*			0.032*	
Fistula output (mL/day)	Fistuloclysis	1306.2 ± 281.1	753.0 ± 254.5	519.6 ± 173.2	786.6 ± 285.8	<0.001*
	Control	1455.2 ± 203.6	1020.5 ± 312.3	1063.7 ± 423.4	391.5 ± 125.3	0.052
	*P* ^b^	0.152			<0.001*	

TB: total bilirubin; DB: direct bilirubin; IB: indirect bilirubin; ALT: alanine aminotransferase; ALP: alkaline phosphatase; GGT: *γ*-glutamyltransferase; TP: total protein; Alb: albumin; WBC: white blood cells; CRP: C-reactive protein. Delta value was defined as the changes from baseline to day 28 for each variable. *P* ^a^ indicated the time-dependent changes of variables within groups. *P* ^b^ indicated comparisons between the fistuloclysis group and the control group (for the baseline value and the delta values, resp.). Data were presented as mean ± SD. *P* value < 0.05 was deemed significant.

**Table 4:** Changes from baseline to day 28 for each variable in the fistuloclysis subgroups.

Variables	Group 1 (*n* = 11)	Group 2 (*n* = 16)	Group 3 (*n* = 8)	*P* value
TB (*μ*mol/L)	13.8 ± 4.1	24.3 ± 8.9	18.4 ± 5.6	0.011*
DB (*μ*mol/L)	10.1 ± 3.2	14.1 ± 5.0	13.2 ± 4.8	0.040*
IB (*μ*mol/L)	3.7 ± 0.8	9.5 ± 3.6	4.3 ± 1.2	<0.001*
ALT (U/L)	10.5 ± 2.5	4.4 ± 1.7	10.3 ± 2.8	<0.001*
ALP (U/L)	77.4 ± 24.9	111.9 ± 32.7	102.6 ± 31.7	0.022*
GGT (U/L)	144.2 ± 50.8	164.2 ± 58.5	158.3 ± 60.3	0.330
TP (g/L)	17.8 ± 6.2	25.7 ± 10.8	21.3 ± 9.8	0.005*
Alb (g/L)	7.5 ± 2.3	14.3 ± 5.4	9.4 ± 3.2	0.020*
WBC (10^9^/L)	2.5 ± 1.1	2.7 ± 1.1	2.6 ± 1.3	0.884
CRP (mg/L)	2.1 ± 0.7	2.8 ± 0.8	2.4 ± 0.7	0.120
Fistula output (mL/day)	755.4 ± 160.2	830.3 ± 231.5	770.0 ± 215.6	0.232

Group 1: patients with jejunal-ileal fistula; Group 2: patients with biliary fistula; Group 3: patients with duodenal fistula. EF: enteric fistula; TB: total bilirubin; DB: direct bilirubin; IB: indirect bilirubin; ALT: alanine aminotransferase; ALP: alkaline phosphatase; GGT:*γ*-glutamyltransferase; TP: total protein; Alb: albumin; WBC: white blood cells; CRP: C-reactive protein. Data were presented as mean ± SD. *P* value < 0.05 was deemed significant.

**Table 5:** Comparison of therapeutic efficacy in the fistuloclysis subgroups.

	Group 1	Group 2	Group 3
	(*n* = 11)	(*n* = 16)	(*n* = 8)
TB (*μ*mol/L)	■	●	
DB (*μ*mol/L)	■	●	
IB (*μ*mol/L)	■	●	
ALT (U/L)	●	■	
ALP (U/L)	■	●	
GGT (U/L)			
TP (g/L)	■	●	
Alb (g/L)	■	●	
WBC (10^9^/L)			
CRP (mg/L)			
Fistula output (mL/day)			
		

Group 1: patients with jejunal-ileal fistula; Group 2: patients with biliary fistula; Group 3: patients with duodenal fistula. TB: total bilirubin; DB: direct bilirubin; IB: indirect bilirubin; ALT: alanine aminotransferase; ALP: alkaline phosphatase; GGT: *γ*-glutamyltransferase; TP: total protein; Alb: albumin; WBC: white blood cells; CRP: C-reactive protein. For each laboratory variable, patients who had the greatest changes were marked with ●. Conversely, patients with the least changes were marked with ■.

## Abbreviations

EN: Enteral nutrition
TPN: Total parenteral nutrition
EF: Enteric fistula
TEN: Total enteral nutrition
TB: Total bilirubin
DB: Direct bilirubin
IB: Indirect bilirubin
APACHE II: Acute physiology and chronic health evaluation II
SOFA: Sequential organ failure assessment
*γ*-GGT: *γ*-Glutamyltransferase
ALP: Alkaline phosphatase
ALT: Alanine aminotransferase
TP: Total protein
WBC: White blood cells
CRP: C-reactive protein
PT: Prothrombin time
APTT: Activated partial thromboplastin time
RBC: Red blood cells
Hb: Hemoglobin.
